# First report of black pustule disease in sponge gourd (*Luffa cylindrica*) in northern Egypt and its biological management

**DOI:** 10.1186/s12870-025-06655-y

**Published:** 2025-05-28

**Authors:** Mohamed Saied Ali Khalil, Nehal Samy El-Mougy, Nadia Gamel El-Gamal, Mokhtar Mohamed Abdel-Kader

**Affiliations:** Plant Pathology Department, National Research Centre, Giza, 12622 Egypt

**Keywords:** *Alternaria alternata*, Bioagents, *Fusarium equesti*, *Luffa cylindrica*, Sponge gourd

## Abstract

**Background:**

In October 2022, black pustules were observed on the lower surface of sponge gourd (*Luffa cylindrica*) leaves in Kafer El-Dawar, North Egypt. Symptoms included abundant black pustules containing fungal conidia on plant leaves, which eventually led to the infected leaves drying out and dying.

**Results:**

Two causal fungi were isolated from symptomatic leaves and their pathogenicity was confirmed to induce typical disease symptoms. On the base on morphological features and further molecular identification, the isolated pathogens were identified as *Alternaria alternata* (accession No. PP197255), and *Fusarium equiseti* (accession No. PP197302). A survey was conducted to detect this disease on luffa plant leaves in northern Egypt, where luffa plants are cultivated. An attempt at biological control of this disease was made for two successive growing seasons under field conditions. BF, algae, *Trichoderma harzianum* and *Bacillus subtilis* were applied as seed treatments, and soil drenches were applied, followed by foliar spraying. Throughout the two seasons, the applied bioagent *B. subtilis* significantly reduced disease severity followed by the *T. harzianum* and algae treatments.

**Conclusion:**

Seed treatment with two bioagents, *T. harzianum* and *B. subtilis*, had the greatest effect on disease severity, followed by soil drenching + foliar spray, soil drench only, and in that respective order. This is the first report of black pustules on the leaves of Luffa plants caused by *Alternaria alternata* and *Fusarium equiseti* in Egypt.

## Background

*Luffa* plants, also known as sponge gourd (*Luffa cylindrica* M. Roem), are subtropical and tropical vegetable plants that belong to the family Cucurbitaceae. *Luffa cylindrica* is an annual climbing plant used in a wide variety of biotechnological applications [1]. They added that Luffa has been successfully used as a matrix for the immobilization of microbiological cells since it was first reported in 1993. The fibro vascular reticulated structure, consisting of an open network of random frameworks with high porosity, low density and high specific pore volume, makes it a suitable carrier for cell immobilization. Loofa-immobilized cell systems have been effectively utilized for treating waste water containing toxic metals, dyes, and chlorinated compounds. This technology has also been used to develop biofilms for remediating domestic and industrial wastewaters rich in inorganic and organic matter. Sponge gourds are primarily grown for their fibrous tissue structure, which is commonly used as brushes, cleaning pads or bath sponges. In addition, immature fruits can be eaten cooked or used as green products in fresh salads [2]. Cucurbits are susceptible to various fungi and are known to be affected by new diseases. Severe leaf spot and fruit rot of *Luffa* caused by *Alternaria* spp. and *Fusarium* spp. have been observed in the field as well as in markets and their control and prevention requires a combination of cultural, biological and chemical strategies were also registered [3–5]. Additionally, Alternaria leaf spot and blight are commonly caused on cucumber plants worldwide by *Alternaria cucumerina* and *Alternaria alternata *[6, 7]. Fungal spores are transported under favorable environmental conditions by long distance winds through rain, warm weather and 60–80% humid conditions.

The target of the current investigation was to identify the causal agents of black pustules noticed on sponge gourd in northern Egypt. This was done on the basis of culture characteristics, molecular phylogenetics, and pathogenicity tests. Additionally, an attempt to suppress the severity of this disease by means of biological control was made under natural field conditions over two successive growing seasons.

## Methods

### Disease occurrence and survey

The total cultivated area of *L. cylindrica*, in Egypt, was determined [8] to be approximately 4,692 feddan, produces 28,700,000 luffa fruits. A field survey was conducted in December 2022 to detect the presence of black pustule disease on luffa plant leaves. The survey took place in different field localities in North Egypt, including Alexandria, Beheira, Kafr El-Sheekh and Sharkia governorates. Ten districts each with ten farms, were selected on the base of their large cultivated area. One hundred randomly selected plants were examined for disease symptoms at each location. The quantity of plants exhibiting disease symptoms was documented, and the incidence percentage was determined in relation to the overall number of plants examined.

### Isolation and identification of the fungi

In October 2022, several Luffa plant leaves exhibiting symptoms with black pustules were collected. The diseased leaf tissue was removed and the surface was sterilized by immersion in 1.0% sodium hypochlorite (Na OCl) for 1 min. The samples were then rinsed three times with sterilized distilled water, placed on potato dextrose agar (PDA) plates, and incubated at 20 ± 2^0^C for 7 days. The mycelium of each fungal colony was then transferred to potato dextrose agar (PDA) via hyphal tip transfer to prepare mono conidial cultures for subsequent tests. The identification of the two fungal isolates was primarily conducted in accordance with the manual of Barnette and Hunter [9]. The morphological features of the fungi were assessed via a light microscope (Olympus cx41) following a growth period of five days. The two isolated strains were subsequently forwarded to the Biotechnology and Molecular Biology Unit, at the National Research Centre for the purpose of fungal molecular identification. Three days-old fungal culture mycelia were obtained via filtration via filter paper (Whatman No. 1). The total genomic DNA was extracted by adhering to the CTAB protocol outlined by Eida et al. [10]. The extracted DNA was amplified via PCR and sequenced for species identification, with a focus on the internal transcribed spacer region of rRNA (ITS), resulting in trimmed sequences 57 bp in length. The isolated genomic DNA was purified via the isopropanol method as described previously [11]. Fungal isolate DNA was amplified through polymerase chain reaction (PCR) utilizing primers ITS1 (5´- TCC GTA GGT GAA CCT GCG G-3´) and ITS4 (5´TCC TCC GCT TAT TGA TATGC—3´). Then, the resulting DNA sequence was compared against reference and type strains available in public data such as GenBank, utilizing the BLAST program from the National Centre for Biotechnology Information (http://www.ncbi.nlm.nih.gov/BLAST). The sequences were aligned via the Jukes Cantor Model and the isolates were subsequently registered in Gen-Bank.

### Pathogenicity test

To perform the pathogenicity assessment, an inoculum was prepared from two fungal isolates. The isolated fungi were subsequently evaluated individually and in combination for their ability to induce black pustules on *Luffa cylindrical* leaves*.* Mature Luffa leaves, which appeared visually unaffected, were collected and subjected to surface sterilization via 1.0% sodium hypochlorite for two min. Subsequently, the leaves were rinsed with sterilized distilled water and allowed to air dry under aseptic conditions.

The lower surface of the sterilized leaves was sprayed with 50 mL of conidial suspensions from each of the two fungi, which were obtained from 7 – 10—day old plants grown in liquid Czapek Dox at 25 ± 2 °C. A set of Luffa leaves was inoculated by spraying 50 mL of conidial suspension (10^6^ spore ml^−1^) of each fungus separately, as were mixtures containing equal proportions of both fungal isolates. Another set of sterilized leaves was sprayed with sterilized distilled water to serve as control treatment. Ten replicates were utilized for both the fungal inoculation and control treatments. The inoculated and no- inoculated leaves were then placed in desiccators containing water at the bottom and incubated at room temperature 22–25 °C for ten days.

### Source of biofertilizers and bioagent microorganisms

Biofertilizers (plant growth-promoting rizobacteria, PGPR), *Pseudomonas fluorescens* (NRC2041) and *Bacillus subtilis* (NRC313), were obtained from the Culture Collection Unit, in the Department of Soil Microbiology, at the National Research Center, in Cairo, Egypt. Cultures of these microorganisms were grown in nutrient broth medium for 48 h at 27 ± 2 °C, and then centrifuged at 3000 × g for 15 min. The pellet was resuspended in sterile distilled water and the final concentration was adjusted to 10^9^ cfu/ml [12]. A volume of 2 L containing fungal spores or bacterial cell suspensions was used for every 100 m^2^ of cultivated Luffa plants.

The two bioagents used in the present study are considered candidate antagonistic isolates, *Bacillus subtilis* and *T. harzianum*, which were kindly supplied by the Culture Collection Unit, in the Department of Plant Pathology, at the National Research Center, Cairo, Egypt. These specific antagonists were previously isolated from Egyptian soil and demonstrated high antagonistic activity against various phytopathogenic microorganisms in several previous studies by the same authors. The inocula of the antagonists, *B. subtilis* (10^8^ cfu/ml) and *T. harzianum* (10^5^ cfu/ml) were used as cell or spore suspensions, respectively [13]. The alga *Spirulina platensis* was kindly supplied by the Algal Biotechnology Unit, in the Fertilization Technology Department, at the National Research Centre, in Egypt. The tested alga was initially prepared in suspension form and stored in dark bottles at 4 °C until use. The fungicide Switch 62.5WG was used as a comparison treatment in this study. Switch 62.5WG was applied at a concentration of 1 g/L. The active ingredients of the fungicide Switch 62.5 WG are cyprodinil (37.5%) and fludioxoni (25%) (https://www.syngenta.ca/productsdetail/switch).

### Field study design and utilized treatments

Under natural infestation, the field trials were conducted over two consecutive seasons, 2023 and 2024. Each growing season included experimental plots measuring 6 × 6 m and, arranged in two rows. Each row consisted of 3 hills located on the eastern side. On the sowing date, the soil was treated with bacterial biofertilizer inocula, following the methodology outlined by Abdel-Kader and El-Mougy [14]**.** The inocula were incorporated into the upper 10 cm of the soil surface and thoroughly mixed to ensure uniform equal distribution.

On May 1st 2023 and 2024, Luffa seeds (cv *Luffa aegyptiaca*) were sown at a rate of two seeds per hill. Luffa plants were separately sprayed with one of abovementioned materials, and distilled water was used for the control treatment. A sprayer tank of 2 L was utilized for every particular treatment. The luffa plants were sprayed twice: first in mid-June, and second, in mid-August. Disease severity was recorded two weeks after the second spray.

Disease severity was assessed for one hundred randomly selected plants. The average disease severity for all applied treatments was estimated on the base of five categories from 0—5 (1) – no infection; (2) – 1–10%; (3) – 10–25%; (4) – 25–50%; (5) – 50–75% and (5) – more than 75% of the leaf lower surface covered with black pustules. The equation described by [15] was as follows:

$$P=\sum \frac{n\times y}{SN}\times 100\left[\%\right],$$

where: *P* – is the disease severity, *n* –is the number of infected leaves in each category, *y* – is the numerical value of each category, *S* –is the highest rating value and *N* –is the total number of the infected leaves.

At the end of the two growing seasons the average of disease severity and harvested fruit yield for all the treatments were recorded.

During each growing season, the following treatments were applied:

1. The soil was treated only with biofertilizer (500 mL of *P. fluorescens* (NRC2041) and *Bacillus subtilis* (NRC313) prepared mixture per 10 m.^2^)
2. The soil was drenched with biofertilizer followed by foliar spraying with a *T. harzianum* suspension (10.^6^ cfu/mL)
3. The soil was treated with biofertilizer and followed by foliar spraying with a *B. subtilis* suspension (10.^8^ cfu/mL)
4. The soil was treated with biofertilizer and followed by foliar spraying with an *S. platensis* algal suspension
5. Seed dressing with a *T. harzianum* suspension (10.^6^ cfu/mL)
6. Seed dressing with *B. subtilis* suspension (10.^8^ cfu/mL)
7. Foliar spray with only the *S. platensis* algal suspension
8. Foliar spray with the fungicide Switch (1.0 g/L)
9. Untreated control

### Data analysis

CoStat version 6.3.3 (CoHort Software) was used to analyze the obtained results. The data from each growing season were subjected to separate statistical analysis through one-way analysis of variance. The means were subsequently then compared via Duncan’s multiple range tests at a significance level of *P* < 0.05.

## Results

### Disease observation and survey

A survey was conducted to assess the incidence of black pustules in *Luffa cylindrical* plant leaves cultivated across various field locations in northern Egypt. The findings of this survey are presented herein. The data in Table (1) revealed that the highest average disease severity, 27.3% occurred in fields belonging to the Alexandria governorate, followed by 25.4% and 18.8% in Beheira and Kafer El-Sheekh, respectively. A lower disease incidence of 13.5% was found in the Sharqia governorate.

**Table 1:** Percentage of black pustule disease incidence in some governorates in North Egypt

Governorate	Average disease incidence (%)
Alexandria	27.3*a
Beheira	25.4b
Kafer El-Sheekh	18.8c
Sharqia	13.5d

^*^Each figure is the mean of examined 500 plants in each of ten farms throughout one governorate of the total four surveyed governorates; Figures with the same letters in the column are not significantly differed (*P* ≤ 0.05) by Duncan multiple range test

**Fig. 1:**
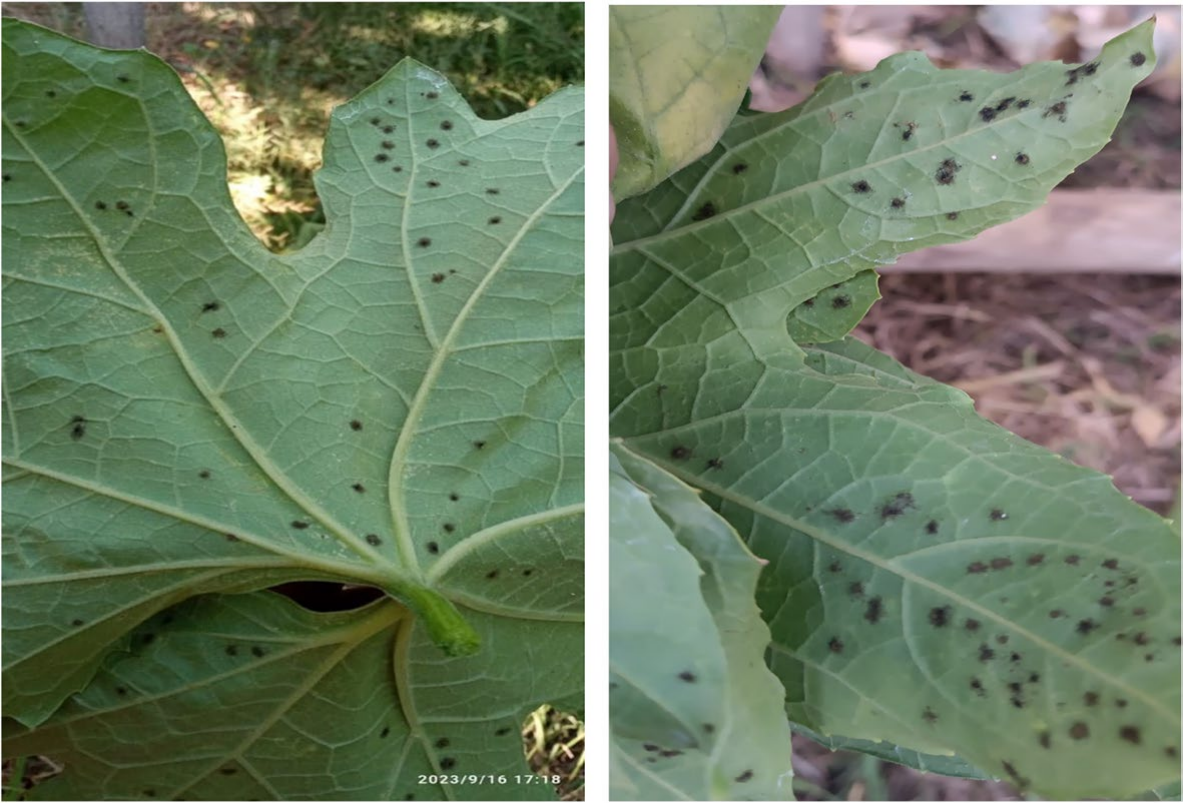
Symptoms of black pustule disease symptoms on the leaves of Luffa (*Luffa cylindrica*)

The disease symptoms initially appeared as separate black swings on the lower surface of Luffa plant leaves. These swellings resembled pustules containing black fungal conidia (Fig. 1). As a consequence of infection, significant areas of the leaves become spoiled, dry and lose their viability.

### Molecular characterization of fungal isolates and their pathogenic capacity

**Fig. 2:**
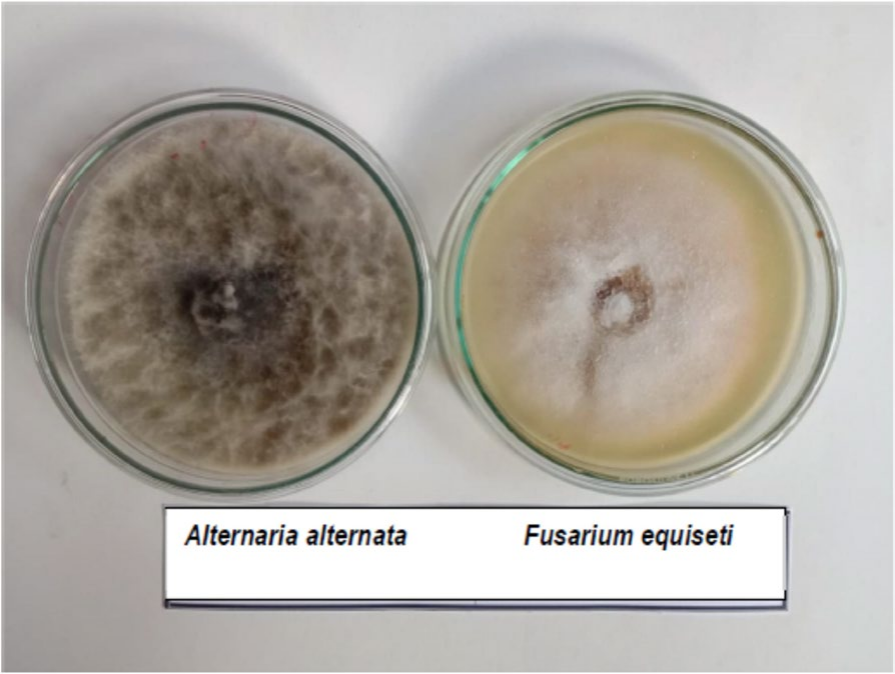
Fungi isolated from black pustules, white color *Fusarium equiseti* and dark gray color for *Alternaria alternata*

Isolations resulted in two fungal isolates on the base of their morphological and microscopic characteristics [9]. The fungal colonies revealed two abundant fungal mycelia growth with white and dark gray conidia (Fig. 2). The morphological and microscopic characteristics revealed showed that the fungal colonies consisted of dense white to yellow felt surrounded by a heavy layer of brown to black conidial heads. Under the microscope, the first identification of the two fungal isolates belonging to the genera *Alternaria* and *Fusarium* was performed. For molecular identification, the obtained sequences were compared to sequences available in GenBank via the BLAST program (http://www.ncbi.nlm.nih.gov/BLAST).

The uniformity of these two isolates according to the sequences submitted to GenBank revealed that the two isolates were *Alternaria alternata* (accession No. PP197255) [Website: https://www.ncbi.nlm.nih.gov/nuccore/PP197255.1?report=genbank], and *Fusarium equiseti* (accession No. PP197302) [website: https://www.ncbi.nlm.nih.gov/nuccore/PP197302.1?report=genbank].

**Fig. 3:**
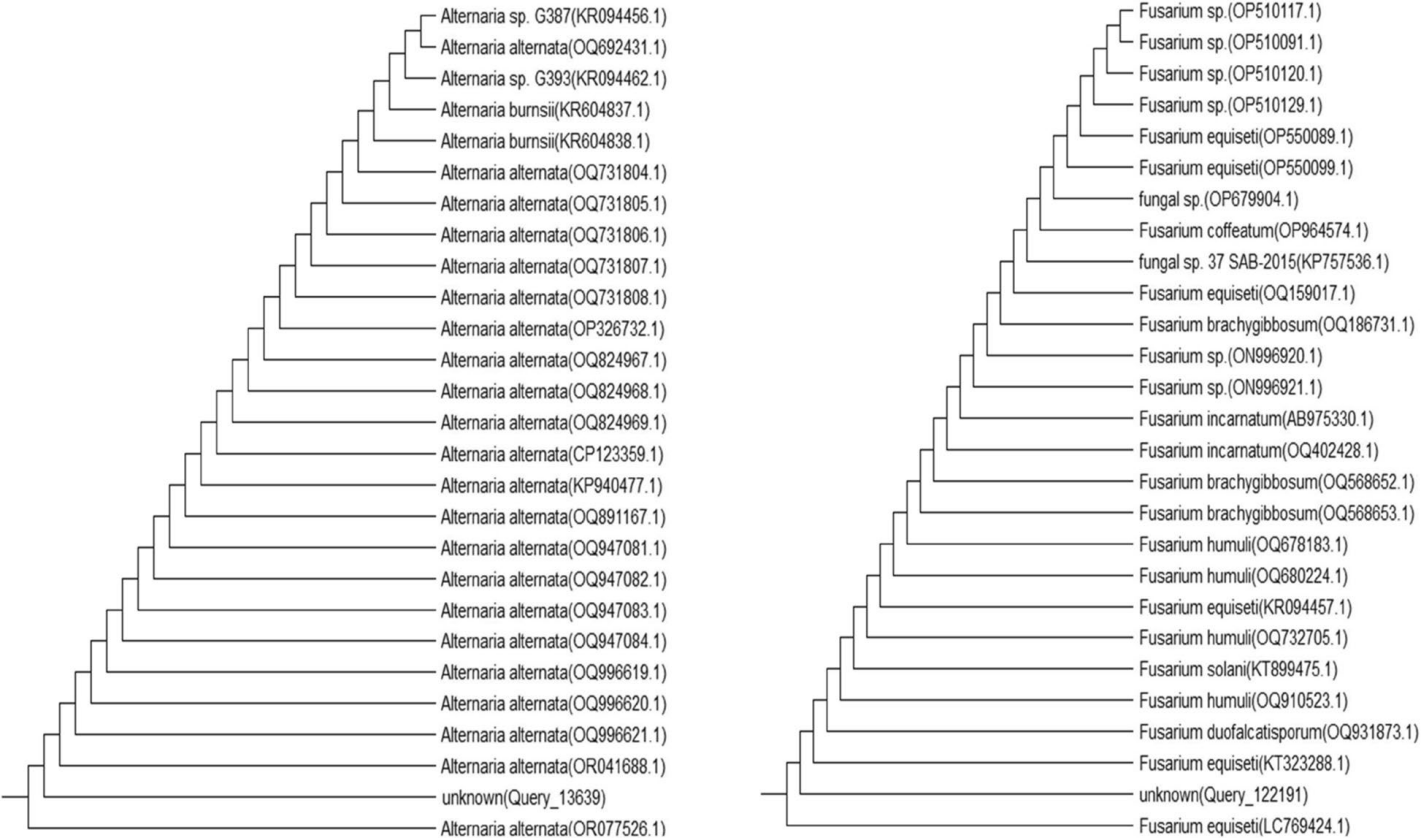
Relationship between the obtained ITS sequences of the two isolated fungal strains of *Alternaria alternata* (on the left) and *Fusarium equiseti* (on the wright) and comparison with those of similar polygenetic strains in GenBank

**Fig. 4:**
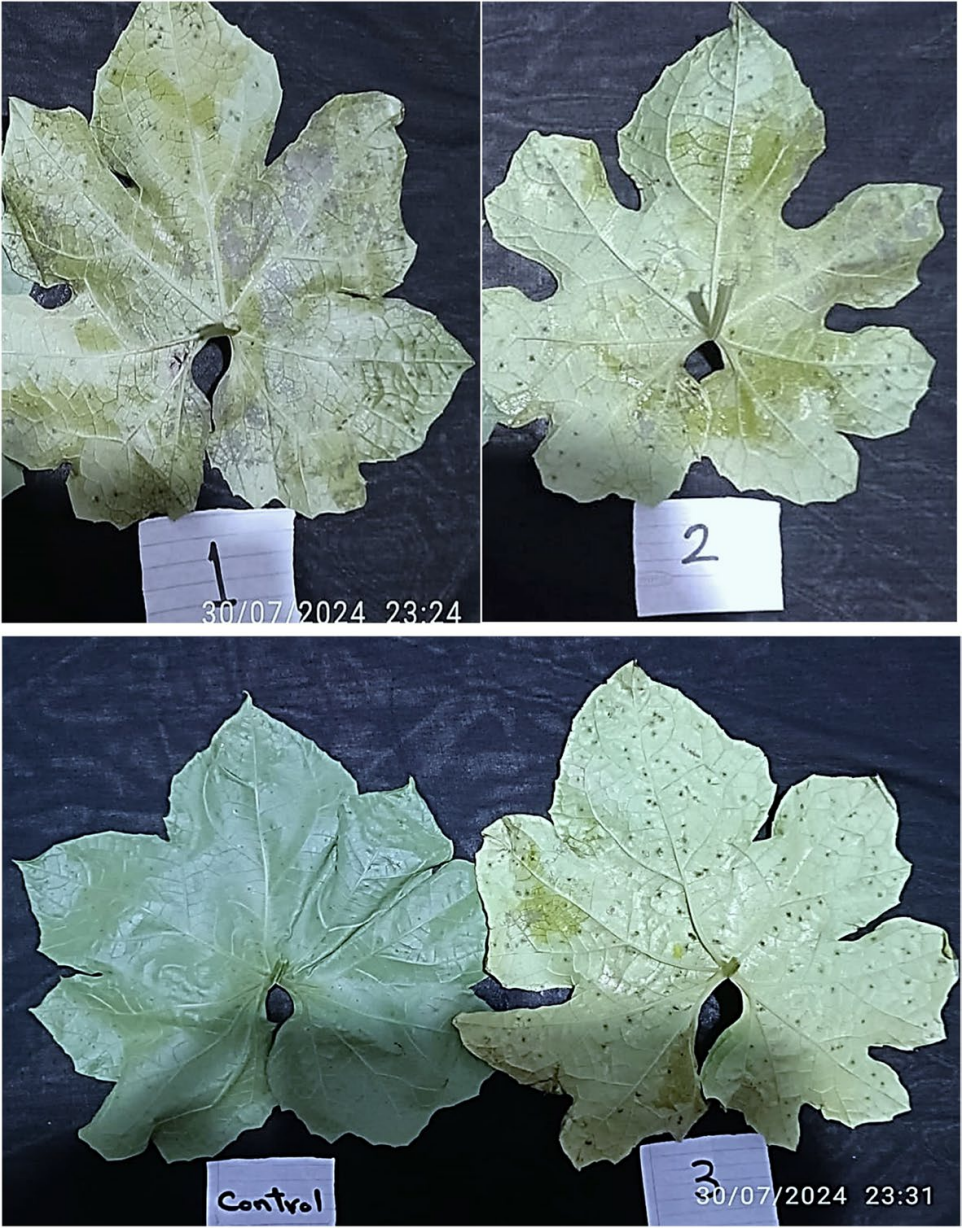
Symptoms of the pathogenic ability of the two tested fungi included the fungal isolate *Alternaria alternata* (1); *Fusarium equiseti* (2) and both fungal isolates (3) to induce black pustule disease on Luffa leaves

The culture collection of the National Centre for Biotechnology Information, which houses a diverse array of fungal species, indicates that the polygenetic relationship between these two isolates is closely aligned with that between the type strains of *Alternaria alternata* and *Fusarium equiseti* (Figs. 3 and 4).

### Field experiments

**Fig. 5:**
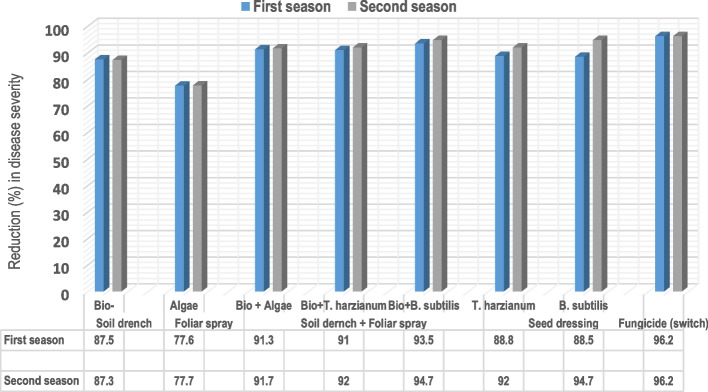
Reduction in disease severity of Luffa black pustules throughout two successive growing seasons in response to various biotreatments

Under natural field conditions, the effectiveness of various biological treatments on Luffa plants was evaluated over two consecutive growing seasons. These treatments include seed dressing, soil drenching, soil drenching with foliar sprays, foliar sprays and fungicide Switch all of which reduce the severity of black pustules on Luffa leaves. Compared with the untreated control, all the treatments effectively reduced disease severity. The data in Table (2) and Fig. (5) indicate that foliar spray with the fungicide Switch was the most effective, by reducing disease severity by 96.2% over the two seasons and resulting in the highest yields of 60.0 and 65.0 fruits, respectively. Soil drenching with biofertilizers plus foliar spraying with *B. subtilis* resulted in a notable reduction in disease severity (93.5%; 94.7%) followed by foliar spraying with *T. harzianum* (91.0%; 92.0%) and the alga *S. platensis* (91.3%; 91.7%). The corresponding yields for these treatments were estimated at (54.0, 60.0); (51.0, 57.0) and (52.0, 58.0) fruits per hundred randomly selected plants. Conversely, the least effective treatments were the application of biofertilizers as soil drenching or seed dressing with *T. harzianum* and *B. subtilis.* These treatments decreased the disease by (87.5%, and 87.3%) and improved the fruits yields by (53.0% and 58.0%) for soil drench with biofertilizers, and (88.8%, and 92.0%) and (88.5%, and 94.7%) for foliar spray. Furthermore, the fruits yields for seed treatments with *T. harzianum* and *B. subtilis* were (50.0, 56.0); (39.0, 43.0), respectively for the two growing seasons.

**Table 2:** Effects of soil drenching and foliar spraying with bioagents on the severity of black pustule disease in Luffa plants under field conditions

Treatment		First season (2023)			Second season (2024)		
		DS (%)^a^	R (%)^b^	Yield^c^	DS (%)^a^	R (%)^b^	Yield^c^
Soil drench							
Bio-fertilizer		5.0 ± 1.2c	87.5	53.0	4.8 ± 1.2c	87.3	58.0
Soil drench + Foliar spray							
Bio- fertilizer	*S. platensis*	3.5 ± 0.8e	91.3	52.0	3.1 ± 1.2d	91.7	58.0
Bio- fertilizer	*T. harzianum*	3.6 ± 1.7e	91.0	51.0	3.0 ± 1.5d	92.0	57.0
Bio- fertilizer	*B. subtilis*	2.6 ± 0.9f	93.5	54.0	2.0 ± 1.3e	94.7	60.0
Seed dressing							
* T. harzianum*		4.5 ± 0.7d	88.8	50.0	3.0 ± 1.5d	92.0	56.0
* B. subtilis*		4.6 ± 1.0d	88.5	39.0	2.0 ± 1.3e	94.7	43.0
Foliar spray							
* S. platensis*		9.0 ± 1.3b	77.6	49.0	8.4 ± 1.9b	77.7	55.0
Fungicide (Switch)		1.5 ± 0.7 g	96.2	60.0	1.4 ± 0.5f	96.2	65.0
Control		40.3 ± 2.4a	-	20.0	37.8 ± 5.0a	-	22.0

*DS*^*a*^ Disease severity, *R*^*b*^ Severity reduction (%) and Yield^c^: Mean No. of fruits per 100 randomly plants

Means ± standard deviations within each column followed by the same letter are not significantly different by Duncan multiple range test at P < 0.05

## Discussion

Luffa plants (*Luffa cylindrica* L. Rox.) are valuable vegetables grown as tropical annual crops cultivated for their multipurpose fruits. In the present study, black pustule disease on the lower surface of Luffa plant leaves was detected in several governorates located in North Egypt. The causal agents were isolated and identified as *Alternaria alternata* and *Fusarium equiseti.* This disease was first observed in the Kafr-El-Dawar governorate in October, 2022, at the end of the growing season. Moreover, this disease seems to have become dominant as it appeared early in May of the two growing seasons of 2023 and 2024. Pathogenicity tests revealed that the two causal fungi could induce disease when inoculated on the lower surface of plant leaves and no disease symptoms were observed when fungal inoculation was performed on the upper surface of the plant leaves. This observation led to the conclusion that the pathogenic fungi invade the plant tissue through the stomata of the leaf. *Alternaria* spp. and *Fusarium* spp. have been previously reported to attack luffa plants causing leaf spots or fruit rot. Downy mildew was first reported [16]. Sponge gourd was reported [17] to be infected by fruit rot caused by *Fusarium oxysporum, Aspergillus niger, Alternaria alternata, Fusarium semitectum* and *Geotrichum candidum.* Alternaria leaf blight on ridge gourd caused by *Alternaria cucumerina* was also reported by [18], in *Luffa cylindrica* caused by *Alternaria temissima* in China. Recently, *Fusarium luffae* has been reported to induce fruit rot on muskmelon [19], flower rot on kiwifruit [20], soybean pod rot [21], and leaf spot on cherry in China [22]. In Egypt, this plant susceptible to a number of various diseases such as powdery mildew [23], and fruit rot [5] which damage plants and cause losses in harvested yield fruits.

The present study focused on biological attempts to suppress disease severity under natural field conditions. Over two successive growing seasons foliar application of the fungicide Switch had the most significant effect on reducing disease severity compared with the untreated control. The fungicide Switch, a combination of cyprodinil and fludioxonil provides effective disease protection on the leaf surface, as well as systemic movement within the plant to target disease. The fungicide is extremely effective against *Botrytis*, *Colletotrichum acutatum*, *Sclerotinia*, *Alternaria* and powdery mildew [24]. Two highly effective active ingredients, Switch® have been reported to control gray mold (*Botrytis*) and other important diseases in brassica, onion, grape, vegetable and strawberry crops. Switch attacks disease pathogens at four different life cycle stages in the pathogen and provide long-lasting control at low use rates [25].

In the following two growing seasons several biological approaches were carried out to suppress black pustules on Luffa leaf plants under natural field conditions. The greatest reduction in disease severity was achieved through soil drench with biofertilizers (PGPR) plus foliar sprays with *B. subtilis*, *T. harzianum,* and the alga *S. platensis*. Moreover, individual treatments with biofertilizer as soil drenches and seed dressings with *B. subtilis* or *T. harzianum* had relatively little effect on decreasing disease severity*.* Plants and bacteria interact in an endless process allowing the plant to absorb nutrients and gain protection with the help of useful bacteria known as plant growth-promoting bacteria (PGPB or PGPR). These bacteria produce bioactive compounds that can help plants to tolerate stress. Plant growth promoting rhizobacteria (PGPR) generate substances such as hormones and help the absorption of specific nutrients, either directly or indirectly. This contributes to the production of antagonistic compounds or strengthens resistance against phytopathogenic microorganisms [26]. According to Pieterse et al. [27], PGPB can act as a catalyst for systemic resistance (ISR), similar to the process of systemic acquired resistance (SAR), which is achieved when host plants defend against pathogen attacks. This ISR (induced systemic resistance) is effective in managing diseases caused by various pathogens. Furthermore, Beneduzi et al. [28] suggested that the resistance induced by Hizobacteria works through the salicylic acid-dependent SAR pathway. They also highlighted that Rhizobacteria from the *Pseudomonas* and *Bacillus* genera are known for their antagonistic properties and their ability to induce ISR. Additionally, Hafez et al. [29] reported a significant decrease in the occurrence and severity of powdery mildew disease in squash plants treated with different bacterial bioagents *Bacillus subtilis*, *B. chitinosporus*, *B. pumilus, B*. *megaterium*, *B. polymexa* and fungal bioagents *T. harzianum*, and *T. viridi*. Additionally, Abd El-Ghany et al. [30] reported that the fungus bioagent, *T. harzianum* and the yeast *Saccharomyces cerevisiae* provided acceptable control of leaf rust disease severity in willow plants. The resistance induction against downy mildew in grapevines was achieved by applying *Trichoderma harzianum* T39 [31].

On the other hand, the algal extract *Spirulina platensis* when applied as a foliar spray or followed by a soil drench with biofertilizer, had a greater effect on reducing black pustule disease on Luffa plant leaves than did the control treatment. Many bioactive secondary metabolites released by marine macroalgae such as antibiotics, antifungals, antivirals and others were reported early [32]. For example, the application of brown alga extract induces plant defense mechanisms in grapevine plants against several pathogens; including *Botrytis cinerea* and *Plasmopara viticola* [33].

Additionally, spraying plants with a commercial extract of the brown seaweed *Ascophyllum nodosum* enhances plant foliar resistance against various carrot and cucumber diseases caused by *Alternaria radicina*, *B*. *cinerea*, *Cladosporium cucumerinum* and *F. oxysporum* [34]. Suppression of root rot fungi; *Fusarium solani* and *Macrophomina phaseolina* in eggplants and watermelon was achieved by the use of extracts of the algae *Polycladia indica* and *Melanothamnus afaqhusainii*, [35]. Additionally, Tiwari and Sharma [36] noted that blue-green algae (cyanobacteria) play an essential role in soil fertility and in controlling agricultural pests and diseases. Moreover, Boualem et al. [37] reported that the characterization of marine macroalgae could referred to their production of biologically active biocidal materials that act against phytopathogens.

In the present study, the harvested fruit yield increased with decreasing disease severity in all the applied treatments.

In this regard, the application of the fungicides Artea and Amistar Xtra caused reduction in powdery mildew, yellow rust and brown rust disease severity and increased significantly the yield of wheat plants under field conditions [38]. A reduction in rice blast disease incidence and an increase in yield when plants are sprayed with *P. fluorescens* have been reported [39]. In a comparable field study conducted by Abdel-Kader et al. [40], the application of a soil treatment with mycorrhizae followed by a plant spray with either *T. harzianum* or *P. fluorescens,* proved to be the most effective method for controlling the severity of powdery mildew, septoria leaf blotch and stem rust diseases in wheat plants. Furthermore, they reported that the obtained yield inversely correlated with the reduction in disease severity. Additionally, the applied fungicide Amistar resulted in a greater reduction in the recorded disease severity than the other tested treatments.

## Conclusions

The results of the present study demonstrated the detection of black pustules on the leaves of Luffa plants. To the best of our knowledge, this report is the first to focus, on *Alternaria alternata* and *Fusarium equiseti* causing black pustules on the leaves of Luffa plants in Egypt. This study provides basic data for the recognition and suppression of this disease. Field approaches with bioagents, biofertilizers, *S. platensis, B. subtilis* and *T. harzianum* at different application methods have been shown to be effective in reducing disease severity. Owing to their wide spectrum activities as antifungal agents they may be considered successful alternatives to classic chemical fungicides for the control of plant diseases.

## Data Availability

The datasets generated and/or analyzed during the current study are available in the [Gen Bank] repository, [Alternaria alternata (accession No. PP197255) [Website: https://www.ncbi.nlm.nih.gov/nuccore/PP197255.1?report=genbank], and Fusarium equiseti (accession No. PP197302) [website: https://www.ncbi.nlm.nih.gov/nuccore/PP197302.1?report = genbank].

## Declarations

This investigation focused on the natural formation of black pustules on Luffa leaves, which has been identified as a new foliar disease, in addition to the utilization of beneficial fungi and bacteria from the environment. Notably, ethical approval was not required for this study.
